# Exosomes derived from M2 macrophages prevent steroid-induced osteonecrosis of the femoral head by modulating inflammation, promoting bone formation and inhibiting bone resorption

**DOI:** 10.1186/s13018-024-04711-1

**Published:** 2024-04-16

**Authors:** Na Yuan, Weiying Zhang, Weizhou Yang, Wenchen Ji, Jia Li

**Affiliations:** 1 Department of Ultrasonography, The First Affiliated Hospital of Xi’an Jiaotong University, Xi’an, Shaanxi Province 710061 China; 2 Department of Orthopedics, The First Affiliated Hospital of Xi’an Jiaotong University, Xi’an, Shaanxi Province 710061 China; 3 Xizang Minzu University, XianYang, Shaanxi Province 712082 China

**Keywords:** Steroid-induced osteonecrosis of the femoral head, Macrophages, Exosomes, Inflammation, Bone resorption, Bone formation

## Abstract

Inflammatory reactions are involved in the development of steroid-induced osteonecrosis of the femoral head(ONFH). Studies have explored the therapeutic efficacy of inhibiting inflammatory reactions in steroid-induced ONFH and revealed that inhibiting inflammation may be a new strategy for preventing the development of steroid-induced ONFH. Exosomes derived from M2 macrophages(M2-Exos) display anti-inflammatory properties. This study aimed to examine the preventive effect of M2-Exos on early-stage steroid-induced ONFH and explore the underlying mechanisms involved. In vitro, we explored the effect of M2-Exos on the proliferation and osteogenic differentiation of bone marrow-derived mesenchymal stem cells(BMMSCs). In vivo, we investigated the role of M2-Exos on inflammation, osteoclastogenesis, osteogenesis and angiogenesis in an early-stage rat model of steroid-induced ONFH. We found that M2-Exos promoted the proliferation and osteogenic differentiation of BMMSCs. Additionally, M2-Exos effectively attenuated the osteonecrotic changes, inhibited the expression of proinflammatory mediators, promoted osteogenesis and angiogenesis, reduced osteoclastogenesis, and regulated the polarization of M1/M2 macrophages in steroid-induced ONFH. Taken together, our data suggest that M2-Exos are effective at preventing steroid-induced ONFH. These findings may be helpful for providing a potential strategy to prevent the development of steroid-induced ONFH.

## Introduction

Steroid-induced osteonecrosis of the femoral head (ONFH) is caused by long-term use of glucocorticoids or short-term use of glucocorticoids in large amounts. With the widespread use of glucocorticoids in clinical practice, the incidence of steroid-induced ONFH is increasing annually, and steroid-induced ONFH has become the most common type of nontraumatic ONFH. Although numerous surgical approaches (core decompression, osteotomies, not vascularized and vascularized bone grafting) are widely described for managing ONFH and preventing progression of ONFH, the results are debated and controversial [1–3].

Clinical and experimental studies have confirmed that inflammatory reactions are involved in the occurrence of steroid-induced ONFH. The levels of TNF-α, a proinflammatory mediator, in the serum and bone marrow of the femoral head were significantly increased in patients and animals with steroid-induced ONFH [4]. Studies have indicated that the treatment of steroid-induced ONFH can be achieved by inhibiting inflammatory reactions. In our previous animal study, pyrrolidine dithiocarbamate (PDTC), an NF-κB inhibitor, was used to inhibit the NF-κB signalling pathway and inflammatory response. PDTC can prevent steroid-induced ONFH by inhibiting osteoclastogenesis and osteocyte apoptosis, and promoting bone formation and angiogenesis [5]. Other studies have shown that in an animal model of femoral head osteonecrosis, inhibiting the proinflammatory factor interleukin-6 can inhibit bone resorbtion and promote bone regeneration and vascular regeneration, thus promoting the repair of osteonecrosis [6, 7]. Therefore, the regulation of the inflammatory response may be an important therapeutic target for treating steroid-induced ONFH.

As the main effector cells of the inflammatory response, macrophages also participate in the occurrence of steroid-induced ONFH [8]. On the one hand, local inflammatory factors in the necrotic femoral head attract macrophages to the necrotic femoral head. On the other hand, the macrophages themselves release inflammatory cytokines such as TNF-α, interleukin-6 and interleukin-12, thereby exacerbating the inflammatory response and further promoting the infiltration of inflammatory cells into the necrotic area [9]. Macrophages are commonly classified into classically activated M1 macrophages and activated M2 macrophages based on their phenotype. M1 macrophages exert proinflammatory activities while M2 macrophages are involved in inflammation resolution. In animal experiments, Wu et al. reported that, in animal experiments, macrophages significantly infiltrated into the necrotic area during the progression of steroid-induced ONFH [8]. In the early stage of osteonecrosis, the main type of macrophages in the necrotic area are M1 macrophages. In the late stage of osteonecrosis, M2 macrophages are the main type of macrophages in the necrotic area. Studies have shown that inhibiting the infiltration of M1 macrophages into the osteonecrotic area and promoting the transformation of M1 macrophages to the M2 macrophage phenotype via interleukin-4 can reduce the inflammatory response and osteocyte apoptosis, and reduce the incidence of osteonecrosis [10].

Extracellular vesicles (EVs) are lipid bilayered vesicles that are secreted from cells towards the extracellular space and play various roles in intercellular communication [11]. EVs consist of exosomes, microvesicles, and apoptotic bodies, categorised based on the size range in diameters. These EVs, specifically exosomes, contain bioactive lipids, proteins, receptors and RNAs, and transmit information from one cell to another by fusing with adjacent cell membranes. EVs exhibit several desirable characteristics including outstanding stability, desirable biocompatibility, extensive biodistribution, and low immunogenicity. These properties make them ideal for therapeutic purposes and regenerative medicine. EVs have shown promise as a therapeutic agent in regenerative medicine, tissue engineering and immunotherapy due to their characteristics that stimulate tissue regeneration and enhance immunomodulatory properties [12–14]. The different cell type affect the contents and functions of EVs [15]. For instance, EVs derived from MSCs have the potential to promote various differentiation pathways, stimulate tissue regeneration, and modulate the immune system [12–14]. However, EVs derived from macrophages carry inflammatory cytokines produced by these cells, which can exert either anti-inflammatory or pro-inflammatory effects [16].

Among EVs, exosomes are attracting increasing attention given their important role in cell-cell communication. Previous studies have demonstrated that exosomes derived from bone marrow mesenchymal stem cells [17], synovial-derived mesenchymal stem cells [18], and human-induced pluripotent stem cell-derived mesenchymal stem cells [19] can prevent steroid-induced osteonecrosis of the femoral head. Furthermore, studies have shown that exosomes derived from M2 macrophages (M2-Exos) have anti-inflammatory properties of parental cells, play an important anti-inflammatory role in the inflammatory response, and promote the formation of an anti-inflammatory microenvironment in the body [20]. M2-Exos were shown to exert protective effects against dextran sulfate sodium-induced colitis [21], myocardial ischemia/reperfusion injury [22] and acute myocardial infarction [23]. However, the effects of M2-Exos on osteonecrosis of the femoral head are unclear. This study was performed to investigate the effect of M2-Exos treatment on ONFH. In addition, we explored the effect of M2-Exos on bone marrow-derived mesenchymal stem cells in vitro and the effect of M2-Exos on inflammation, bone resorption (osteoclastogenesis) and bone formation (osteogenesis and angiogenesis) in vivo to identify the underlying mechanisms involved.

## Materials and methods

### Culture of macrophages and macrophage polarization

Bone marrow derived macrophages were isolated as described previously [24, 25]. The studies were approved by the Animal Ethical Committee of Xi’an Jiaotong University and complied with the Guide for the Care and Use for Laboratory Animals published by the US National Institutes of Health. Six SD rats were sourced from the Experimental Animal Center of Xi’an Jiaotong University (Shaanxi, China) and used for isolation of bone marrow derived macrophages. After the SD rats were killed and soaked in 75% ethanol for 10 min, the femurs and tibias bones were isolated under sterile conditions. Bone marrow was flushed into α-minimum essential medium (αMEM) supplemented with 10% fetal bovine serum (FBS, Gibco, CA, USA) and 1% penicillin-streptomycin (Gibco, CA, USA) by repeated aspiration with 10 ml syringes. The cell suspension was centrifuged at 500 × g for 5 min, and the pellet was resuspended in erythrocyte lysate to lyse red blood cells for 5 min. The cells were washed with αMEM and cultured in αMEM supplemented with 10% FBS, 1% penicillin-streptomycin and 10 ng/ml monocyte colony stimulating factor (M-CSF) for 7 days to allow them to differentiate into M0 macrophages. For M2 polarization, the macrophages were cultured in medium containing 20 ng/mL interleukin (IL)-4 (Sigma-Aldrich, St Louis, MO, USA). After 24 h of induction, the macrophage phenotypes were identified by using flow cytometry (FCM) and quantitative reverse transcription polymerase chain reaction (RT-qPCR) analysis of M2-related markers.

### Flow cytometry

The M2 macrophage phenotypes were identified by FCM using antibodies against CD206. Briefly, the cells were incubated with an anti-CD206 antibody (Invitrogen) for 30 min at 4 °C. For immunolabeling, the cells were then incubated with a goat anti-rabbit IgG (H + L) cross-absorbed secondary antibody, FITC (Invitrogen), for 30 min at 4 °C in the dark. Excess antibody was removed by washing the cells with PBS. Then, the cells were analysed by flow cytometry on a FACSCalibur system.

### Isolation and identification of exosomes

Exosomes were purified from M2 macrophages by differential ultracentrifugation as previously described [26]. Briefly, the conditioned medium was centrifuged at 300 × g for 10 min and 16,500 × g for 20 min to remove debris and dead cells, followed by filtration through a 0.22 μm filter. Then, the exosomes were pelleted by ultracentrifugation at 120,000 × g for 70 min at 4 °C, resuspensed in PBS and stored at -80 °C for subsequent experiments. The morphology (cup-shaped) of the exosomes was examined using transmission electron microscopy(TEM). The size distribution of the exosomes was verified using a NanoSight NS300 (Marvel, UK). To determine the typical surface markers of exosomes, including CD9 and TSG101, Western blotting was performed.

### Isolation and culture of bone marrow-derived mesenchymal stem cells

Rat bone marrow-derived mesenchymal stem cells were isolated as described previously [26]. Three SD rats were used in this study. Briefly, the femurs and tibias were resected under sterile conditions. Bone marrow was flushed out by repeated aspiration with syringes. After centrifugation at 800 rpm for 5 min, the collected cells were seeded into 6-well dishes and cultured in αMEM supplemented with 10% fetal bovine serum (FBS, Gibco, CA, USA) and 1% penicillin-streptomycin (Gibco, CA, USA) in an incubator with 5% CO_2_ at 37 °C. Cells at passages 3–5 were used in subsequent experiments.

### Cell proliferation assay

Cell Counting Kit-8 (CCK-8) assays (Dojindo, Kumamoto, Japan) were used to assess the effects of M2-Exos on the proliferation of BMMSCs. BMMSCs were seeded into 96-well plates at a density of 2 × 10^3^ cells/well and treated with culture medium containing M2-Exos (50 µg/mL) or PBS at the same volume for 2 days. Ten microlitres of CCK-8 solution was added to each well. After incubation for another 3 h, the absorbance was measured at 450 nm with a spectrophotometer.

### Alkaline phosphatase (ALP) activity assay, ALP staining and Alizarin Red staining(ARS)

To assess the effects of M2-Exos on osteogenic differentiation of BMMSCs, ALP activity assays, ALP staining and Alizarin Red staining were performed according to a previously reported protocol [27]. BMMSCs were cultured in osteogenic medium (α-MEM containing 10% exosome-free FBS, 10 nM dexamethasone, 50 µM L-ascorbic acid-2-phosphate, and 10 mM β-glycerophosphate; all from Sigma) supplemented with M2-Exos (50 µg/mL), or the same volume of PBS. After 7 days, the ALP activity was determined using p-nitrophenyl phosphate (p-NPP, Sigma) as a substrate and quantified colorimetrically at 405 nm. After 7 days of osteogenic induction, ALP staining was performed to analyse the osteoblastic differentiation. After 14 days, alizarin red staining was performed. For the quantitative assessment of alizarin red staining, the absorbance at 590 nm was detected using a spectrophotometer.

### Quantitative reverse transcription polymerase chain reaction analysis (RT-qPCR)

Total RNA was extracted from cells using TRIzol reagent (Invitrogen). cDNA was synthesized by using a PrimeScript RT kit (Takara Bio, Dalian, China). RT-qPCR was performed with an ABI 7500 system (Applied Biosystems). GAPDH mRNA levels were used for normalization. Relative gene expression levels were calculated by the comparative 2^−ΔΔCt^ method. The primers used were as follows: 5ʹ-GCCAGCCTCGTCTCATAGACA-3ʹ (forwards), 5ʹ-AGAGAAGGCAGCCCTGGTAAC-3ʹ (reverse) for GAPDH; 5ʹ-CGAATAACAGCACGCTATTAA-3ʹ (forwards), 5ʹ-GTCGCCAAACAGATTCATCCA-3ʹ (reverse) for RUNX2; 5ʹ-ACCCTCTCTCTGCTCACTCTGCT-3ʹ (forwards), 5ʹ-GCTCCAACTCCATTGTTGAGGTAG-3ʹ (reverse) for OCN; 5ʹ-AGGGTTACTTGGGTTGCC-3ʹ (forwards), 5ʹ-GGGTCTTCAGCTTCTCTCC-3ʹ (reverse) for IL-10; 5ʹ-CAGTATTCACCCCGGCTA-3ʹ (forwards), 5ʹ-CCTCTGGTGTCTTCCCAA-3ʹ (reverse) for Arg-1; 5ʹ-TGTTTTGGCTGGGACTGACCTA-3ʹ (forwards), 5ʹ-CGGGTGTAGGCTCGGGTAGTAG-3ʹ (reverse) for CD206.

### Establishment of a rat model of steroid-induced ONFH

Sixty healthy male SD rats (age: 8–10 weeks; body weight: 260–320 g) were randomly divided into three groups (*n* = 20 per group): the Normal group, Model group and M2-Exos group. The rat steroid-induced ONFH model in an early stage was established by using lipopolysaccharide combined with methylprednisolone according to published protocol [28]. Rats in Model group and M2-Exos group were intravenously injected twice with 4 mg/kg lipopolysaccharide (LPS, Sigma, St. Louis, MO, USA) at a time interval of 24 h. Twenty-four hours later, the animals were intramuscularly injected with 60 mg/kg methylprednisolone (MPS, Pfizer, New York, USA) once a day for 3 days. Rats in Normal group were treated with the same volume of saline buffer as those in Model group. Rats in M2-Exos group were intravenously injected with 100 µg of exosomes derived from M2 macrophages every week for 4 weeks. The dosage and timing of M2-Exos was based on references from previous literature [26, 29, 30]. At the same time, rats in Model group were intravenously injected with an equal volume of PBS. Four weeks after the last injection of MPS, the rats were randomly selected for further study.

### Hematological examination

Four weeks after the last injection of MPS, blood sample was obtained. The levels of inflammatory markers including TNF-α, IL-6 and IL-10 were quantified by commercially available ELISA kits (R&D Systems, USA)according to the manufacturer’s instructions. The optical density was measured at 450 nm using a spectrophotometer (Thermo). Then, concentrations, expressed as pg/mL, were calculated according to the optical density and the standard curve.

### Hematoxylin-eosin (HE) staining

Four weeks after the last injection of MPS, ten rats of each group were euthanized and the femoral heads were collected from both sides. The left samples were fixed in 10% neutral buffered formalin for one week, decalcified with 10% EDTA for four weeks, dehydrated through graded ethanol solutions, embedded in paraffin, cut into 4-µm sections and stained with hematoxylin-eosin. The osteonecrotic changes in every group were observed by two experienced observers using a light microscope in a blinded fashion. The evaluation criteria for osteonecrosis in this model was based on previous reports [31, 32]. Osteonecrosis was judged according to the presence of empty lacunae or pyknotic nuclei of osteocytes within the trabecular bone, along with the accumulation of hypertrophy fat cells and debris of bone marrow cells. The rate of empty lacunae was calculated in five randomly selected fields per section. The right samples were stored for Micro-CT Scanning.

### Immunohistochemical staining and TRAP staining

To evaluate osteogenic and adipogenic activity, immunohistochemical staining for osteocalcin(OCN) and peroxisome proliferator-activated receptor γ (PPAR-γ) was conducted. In addition, to evaluate angiogenesis, immunohistochemical staining for vascular endothelial growth factor(VEGF) was performed. Furthermore, to assess inflammatory reactions, immunohistochemical staining for TNF-α, IL-6 and IL-10 was performed. Images were quantitatively analyzed with Image-Pro Plus Software. The mean density was calculated(defined as the ratio of integrated optical density to total area).

To assess osteoclast activity, tartrate-resistant acid phosphatase (TRAP) staining was conducted using a TRAP staining kit from Sigma-Aldrich. TRAP-positive cells were labelled with purplish red. Five sections were taken from each rat and ten optical fields (magnification: ×100) in each specimen were randomly selected for the evaluation of number of osteoclasts. Active osteoclasts were defined as TRAP-positive multinuclear cells containing three or more nuclei.

### Double fluorescence labelling of tetracycline and calcein

Five rats were injected subcutaneously with tetracycline hydrochloride (25 mg/kg, Sigma-Aldrich, St. Louis, MO, USA) to mark sites of bone formation on the 14th and 13th days before sacrifice. Ten days later, the rats were injected subcutaneously with the second bone marker calcein (10 mg/kg, Sigma-Aldrich, St. Louis, MO, USA) on the 4th and 3rd days before sacrifice. Then, the samples were collected, fixed in 10% formalin solution, embedded in methyl methacrylate and polished. The sections were visualized and photographed under a fluorescence microscope. The mineral apposition rate (MAR, µm/d) was calculated by dividing the tetracycline (yellow) and calcein (green) double-labelling interval by the administration time interval(10 days).

### Micro-CT scanning

Four weeks after the final injection of MPS, the femoral heads were dissected from the rats and scanned by high-resolution micro-CT (eXplore Locus SP, GE, USA) with a resolution of 14 μm per pixel. Three-dimensional (3D) reconstruction was accomplished using a Reconstruction Utility. The region of interest (ROI) was selected in the femoral head without the cortical shell and quantitative parameters including bone mineral density (BMD), bone volume/total volume of bone (BV/TV), trabecular number (Tb.N), and trabecular separation (Tb.Sp) were calculated to evaluate the microarchitecture of the trabecular bone in the femoral heads.

### Angiography

Micro-CT-based angiography was performed in five rats to detect the blood supply of the femoral head as previously reported [26]. After anesthesia, the abdominal aorta and vein were dissected. Microfil MV-122(Flow Tech, Carver, MA, USA) was perfused into the distal abdominal aorta to visualize vessels within the femoral head. After being placed at 4 °C for 24 h, the bilateral femoral heads were obtained and decalcified in 10% EDTA for four weeks until the femoral heads could be easily pierced with a pin. Then, the samples were scanned by micro-CT as described above. Three-dimensional reconstruction of blood vessels was accomplished, and the vascular volume was calculated.

### Immunofluorescence staining

To study M1 and M2 macrophage density, sections were analysed for the specific marker of the M1 phenotype (CD86) and the marker of the M2 phenotype (CD206) by immunofluorescence staining. Briefly, the sections were incubated with anti-CD86 (1:200; Abcam) and anti-CD206 (1:1000; Abcam) primary antibodies at 4 °C overnight, followed by staining with a fluorescein-conjugated antibody (1:200, DAKO) for 1 h at room temperature. Then, the sections were treated with 4,6-diamino-2-phenylindole (DAPI) to stain the nuclei. All images were obtained using a fluorescence microscope(Olympus). Quantitative analysis of CD86-positive macrophages (M1) and CD206-positive macrophages (M2) in each group was performed by two researchers who were blind to the groups. The number of M1 and M2 phenotype macrophages was counted at 200× magnification by selecting ten random fields per slide. Based on five independent replicates of each group, the percentages of M1 and M2 phenotype macrophages were calculated as the percentages of CD86-positive cells/total DAPI-positive cells and CD206-positive cells/total DAPI-positive cells, respectively.

### Statistical analysis

All data were analysed by SPSS 18.0. Categorical data were analysed using Pearson’s chi-square test. All the numerical data are shown as the mean ± standard deviation (SD). Student’s t test was used to compare numerical data between two groups, and one-way analysis of variance (ANOVA) was used to compare numerical data among multiple groups. *p* < 0.05 was considered statistically significant.

## Results

### Identification of macrophage phenotypes and exosomes

**Fig. 1:**
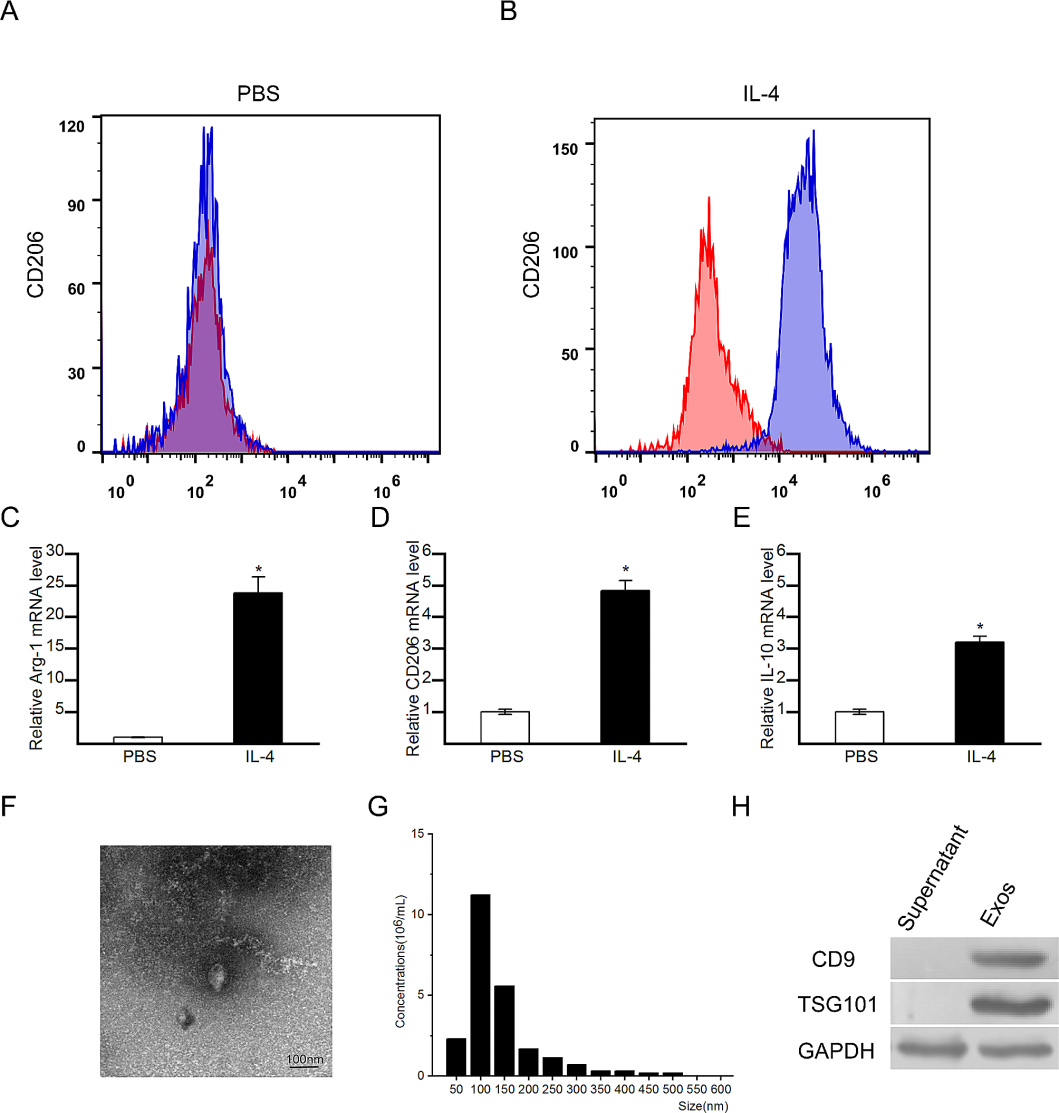
Identification of M2 macrophages and exosomes. (**A**) Flow cytometry analysis of CD206(M2 marker) in PBS-treated macrophages. (**B**) Flow cytometry analysis of CD206(M2 marker) in IL-4-treated macrophages. (**C-E**) Expression levels of Arg-1, CD206 and IL-10(M2-specific markers) in PBS-treated or IL-4-treated cells measured by qRT-PCR. (**F**) Morphology of exosomes derived from M2 macrophages observed by TEM. (**G**) Size distribution of exosomes detected by nanoparticle tracking analysis. (**H**) Detection of exosome surface markers by Western blotting. The data are presented as the means ± SDs; *N* = 5; *p < 0.05

After the rat bone marrow derived macrophages were isolated and stimulated with IL-4 for M2 polarization, the expression of M2 surface markers on macrophages was analysed by flow cytometry. Compared to those of PBS-treated macrophages, the cells treated with IL-4 showed significant upregulation of CD206 (a specific surface marker of M2 macrophages, Fig. 1A and B). The mRNA expression levels of M2-specific genes were quantified by qRT-PCR. Compared with those of cells treated with PBS, cells treated with IL-4 had significantly upregulated expression levels of Arg-1, CD206 and IL-10 (M2-specific markers) (Fig. 1C, D and E). All of these results suggested that the M2 polarization was successfully induced by IL-4.

The exosomes were extracted from the M2 macrophages by ultracentrifugation. TEM images and nanoparticle tracking analysis revealed that exosomes derived from M2 macrophages exhibited classic cup-shaped morphology with a typical diameter of 40–150 nm(Fig. 1F and G). Furthermore, the expression of exosome markers, including CD9 and TSG101 was detected by Western blotting(Fig. 1H). These data indicated that the exosomes were successfully isolated from the M2 macrophages.

### M2-Exos promoted the proliferation and osteogenic differentiation of BMMSCs

**Fig. 2:**
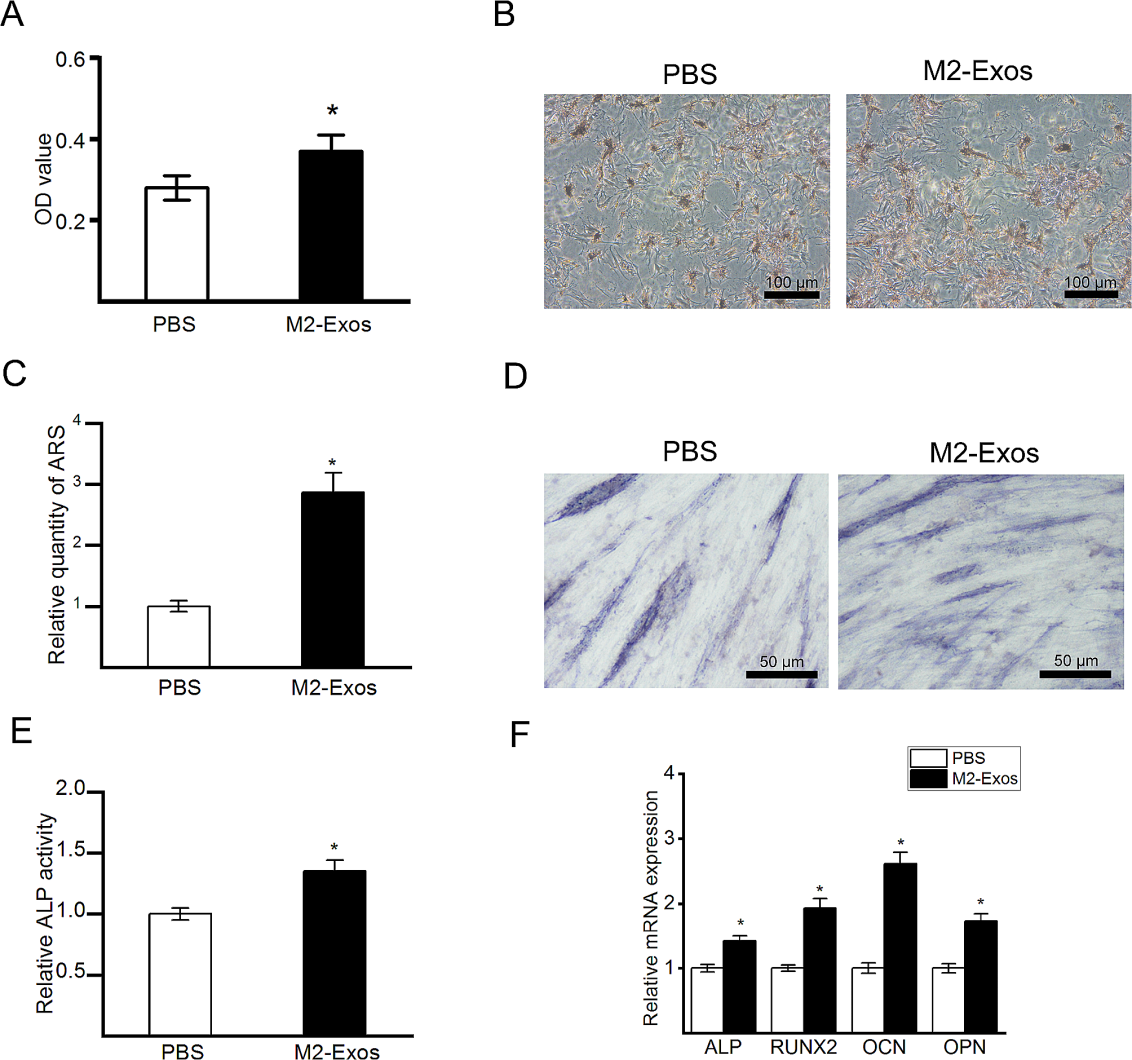
The effects of M2-Exos on the proliferation and osteogenic differentiation of BMMSCs. (**A**) Proliferative potential of BMMSCs indicated by the CCK-8 assay. (**B**) Representative images of Alizarin red staining. (**C**) Quantitative analysis of alizarin red staining. (**D**) Representative images of ALP staining. (**E**) Quantitative analysis of ALP activity. (**F**) Expression of ALP, RUNX2, OCN and OPN mRNA measured by qRT-PCR. The Data are presented as the means ± SDs; *N* = 5; *p < 0.05

Rat BMMSCs were isolated and cultured with M2-Exos to study the effects of M2-Exos on the proliferation and osteogenic differentiation of BMMSCs. To investigate the effect of M2-Exos on the proliferation of BMMSCs, a CCK-8 assay was performed. The data from the CCK-8 assay (Fig. 2A) showed that the supplementation with M2-Exos obviously promoted the proliferation of BMMSCs. We next investigated the effect of M2-Exos on the osteogenic differentiation of BMMSCs. ALP staining and ALP activity assays were conducted on Day 7, and Alizarin red staining was conducted on Day 14. The results of Alizarin red staining (Fig. 2B) showed that BMMSCs cultured with M2-Exos had increased calcium deposition. Quantitative analysis of Alizarin red staining (Fig. 2C) indicated that M2-Exos significantly promoted the mineralization of BMMSCs. ALP staining (Fig. 2D) and the ALP activity assay (Fig. 2E) showed similar results. The results of the ALP activity analysis revealed that BMMSCs cultured with M2-Exos showed increased ALP activity compared with that of BMMSCs cultured with the control treatment. In addition, the mRNA expression of osteogenesis-related genes, including ALP, RUNX2, OCN and osteopontin (OPN), was examined by qRT-PCR on Day 14. The results (Fig. 2F) indicated that M2-Exos increased the mRNA levels of ALP, RUNX2, OCN and OPN.

### M2-Exos attenuated the osteonecrotic changes in the rat ONFH model

**Fig. 3:**
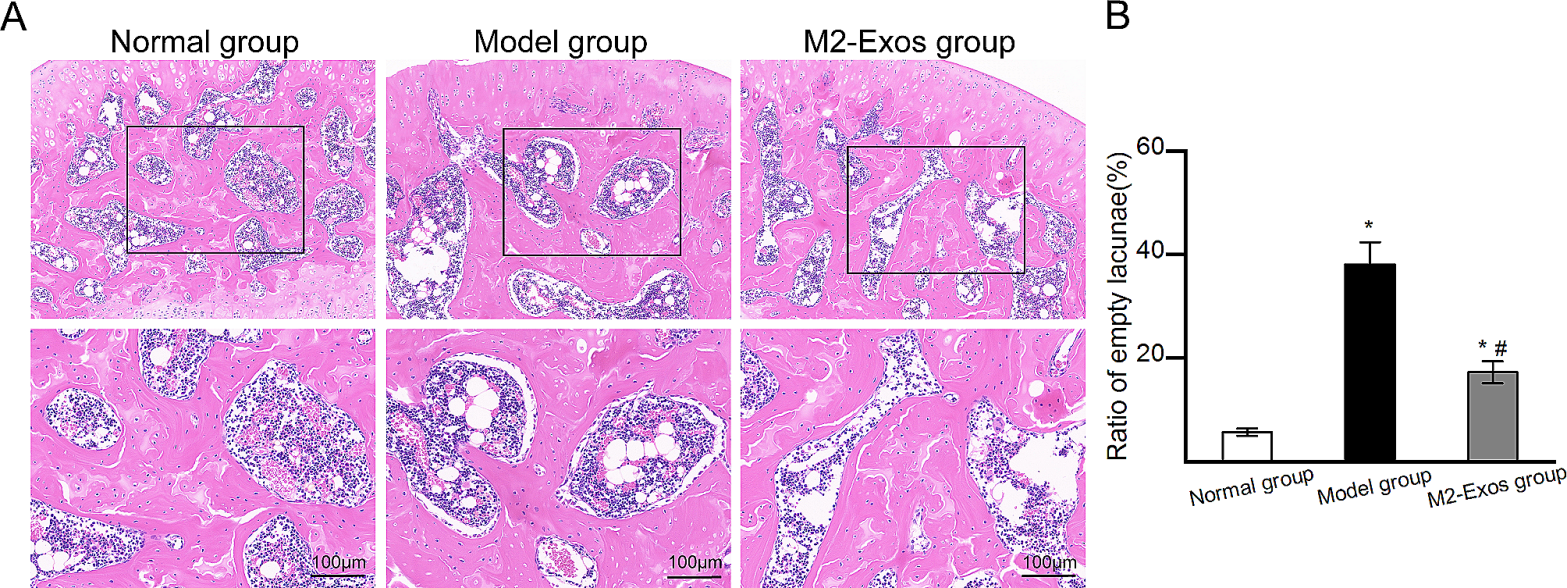
Histopathological changes in the femoral heads of the different groups by hematoxylin-eosin staining. (**A**) Representative images of hematoxylin-eosin staining. Black blocks indicate the magnified area. (**B**) The ratio of empty lacunae in different groups. *N* = 5 per group.*p < 0.05 compared with Normal group; #*p* < 0.05 compared with Model group

To investigate the effects of M2-Exos on steroid-induced ONFH, the histopathological changes in femoral heads were observed(Fig. 3A and B). No osteonecrotic changes were observed in Normal group. In Model group, increased empty lacunae along with the accumulation of hypertrophy fat cells and debris of bone marrow cells were observed. In M2-Exos group, the osteonecrotic changes were attenuated, and the trabeculae showed better structural integrity with reduced numbers of empty lacunae. Compared with that in Model group, M2-Exos group exhibited an obviously decreased ratio of empty lacunae(Fig. 3B).

### Effects of M2-Exos on inflammatory mediators

**Fig. 4:**
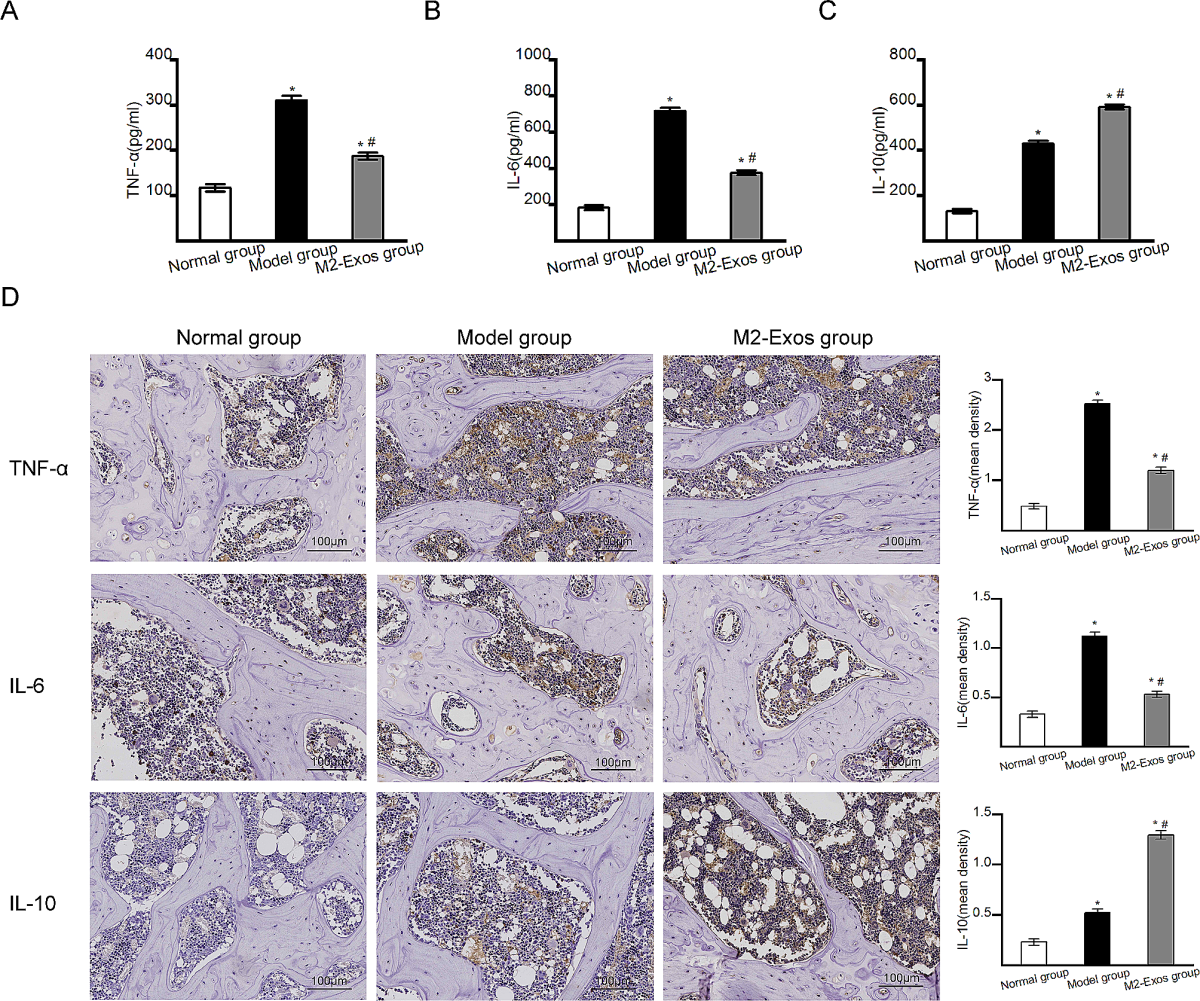
The expression of inflammatory mediators in different groups. (**A**) Serum expression level of TNF-α measured by ELISA. (**B**) Serum expression level of IL-6 measured by ELISA. (**C**) Serum expression level of IL-10 measured by ELISA. (**D**) The expression of TNF-α, IL-6 and IL-10 in the femoral heads of the different groups detected by immunohistochemical staining. Bar graphs showed the mean optical density of TNF-α, IL-6 and IL-10. *N* = 5 per group. *p < 0.05 compared with Normal group; #*p* < 0.05 compared with Model group

Inflammatory factors play important roles in the occurrence of steroid-induced ONFH. To explore the effects of M2-Exos on inflammatory mediators, the expression of inflammatory markers including TNF-α, IL-6 and IL-10, was detected by ELISA kits and immunohistochemical staining. As shown in Fig. 4A-C, the serum expression levels of both proinflammatory factors (TNF-α and IL-6) and an anti-inflammatory factor (IL-10) were significantly higher in Model group than in Normal group at 4 weeks. The expression levels of proinflammatory factors (TNF-α and IL-6) were lower in M2-Exos group than in Model group, whereas the expression levels of an anti-inflammatory factor (IL-10) were higher in M2-Exos group than in Model group. The immunohistochemical staining results (Fig. 4D) showed the similar results for the femoral head. These results suggested that the elevation of proinflammatory mediators in steroid-induced ONFH and M2-Exos administration ameliorated inflammatory responses.

### M2-Exos promoted osteogenesis and angiogenesis in vivo

**Fig. 5:**
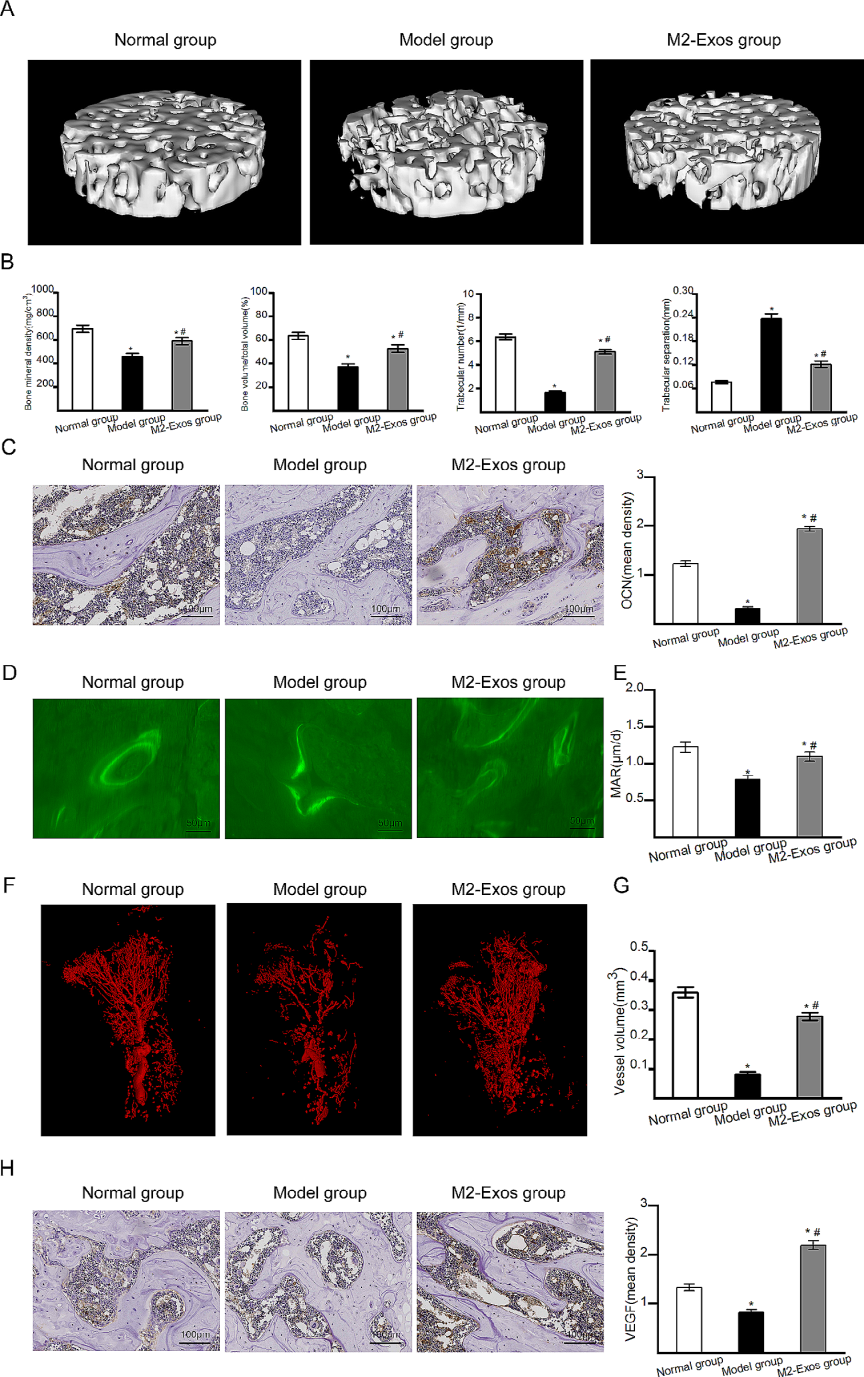
Osteogenesis and angiogenesis in vivo. (**A**) Representative three-dimensional reconstructed micro-CT images. (**B**) Quantitative analysis of micro-CT scanning. (**C**) Immunohistochemical staining of OCN. Bar graph showed the mean optical density of OCN. (**D**) New bone formation indicated by double fluorescence labelling with tetracycline and calcein. (**E**) Mineral apposition rate in different groups. (**F**) Representative images of micro-CT-based microangiography. (**G**) Quantitative analysis of vessel volume. (**H**) Immunohistochemical staining of VEGF. Bar graph showed the mean optical density of VEGF. *N* = 5 per group. *p < 0.05 compared with Normal group; #*p* < 0.05 compared with Model group

The trabecular bone morphology of the femoral heads was analysed by micro-CT scanning. The results (Fig. 5A) showed that compared with that in Model group, M2-Exos group exhibited better structural integrity. Quantitative analysis (Fig. 5B) of micro-CT scanning indicated that M2-Exos group exhibited superior microstructural parameters including BMD, BV/TV, Tb.N, and Tb.Sp. The expression of the osteogenic factor OCN was explored by immunohistochemical staining. As shown in Fig. 5C, the expression of OCN was lower in Model group than in Normal group. Compared with that in Model group, the expression of OCN in M2-Exos group increased after M2-Exo administration. Bone formation was determined by double fluorescence labelling of tetracycline and calcein (Fig. 5D). The results of the quantitative analysis (Fig. 5E) showed that there was a significantly higher mineral apposition rate in M2-Exos group than in Model group. To analyse angiogenesis in the femoral head, micro-CT-based microangiography and immunohistochemical staining for VEGF were conducted. The results of micro-CT-based microangiography (Fig. 5F) indicated that Model group exhibited impaired vessel structure compared with that in Normal group and M2-Exos group exhibited better vessel structure compared with Model group. The results of the quantitative analysis (Fig. 5G) showed that M2-Exos group had significantly increased vessel volume compared with Model group. Immunohistochemical staining for VEGF (Fig. 5H) revealed similar results. Taken together, these results indicated that M2-Exos promoted osteogenesis and angiogenesis in steroid-induced ONFH.

### M2-Exos reduced osteoclastogenesis and adipogenesis in vivo

**Fig. 6:**
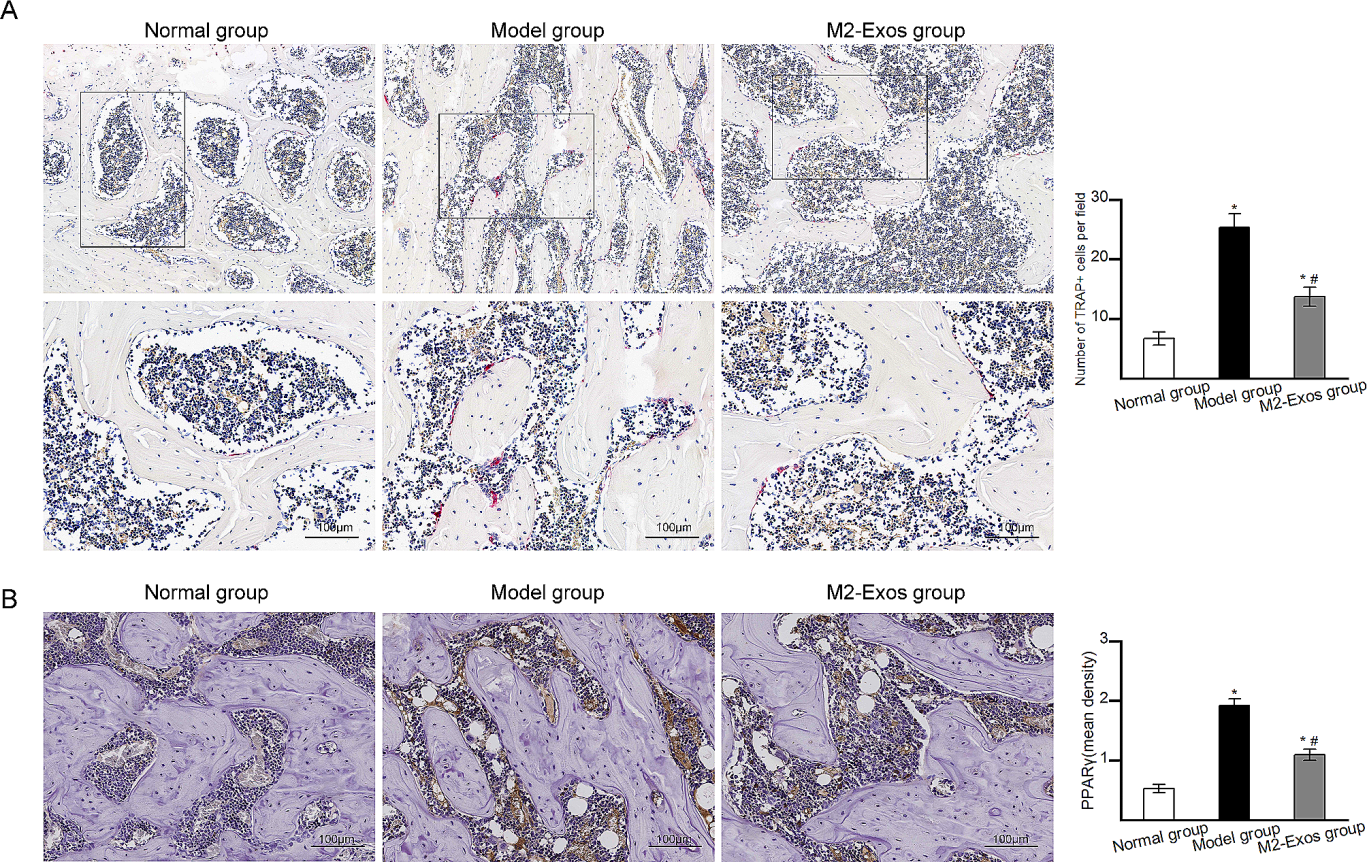
Osteoclastogenesis and adipogenesis in vivo. (**A**) Representative images of TRAP staining (the osteoclasts were stained wine red). Black blocks indicate the magnified area. (**B**) Immunohistochemical staining of PPARγ. Bar graph showed the mean optical density of PPARγ. *N* = 5 per group. *p < 0.05 compared with Normal group; #*p* < 0.05 compared with Model group

TRAP staining was performed to determine the number of osteoclasts in the femoral head in different groups. TRAP staining (Fig. 6A) revealed a significantly higher number of osteoclasts in Model group than in Normal group. The M2-Exos group had a significantly lower osteoclast number than Model group. The expression of PPARγ, which plays a vital role in modulating adipogenesis, was explored by immunohistochemical staining. As shown in Fig. 6B, the expression of PPARγ was higher in Model group than in Normal group. M2-Exos treatment decreased the expression of PPARγ in M2-Exos group compared with that in Model group.

### Effects of M2-Exos on the polarization of M1/M2 macrophages in vivo

**Fig. 7:**
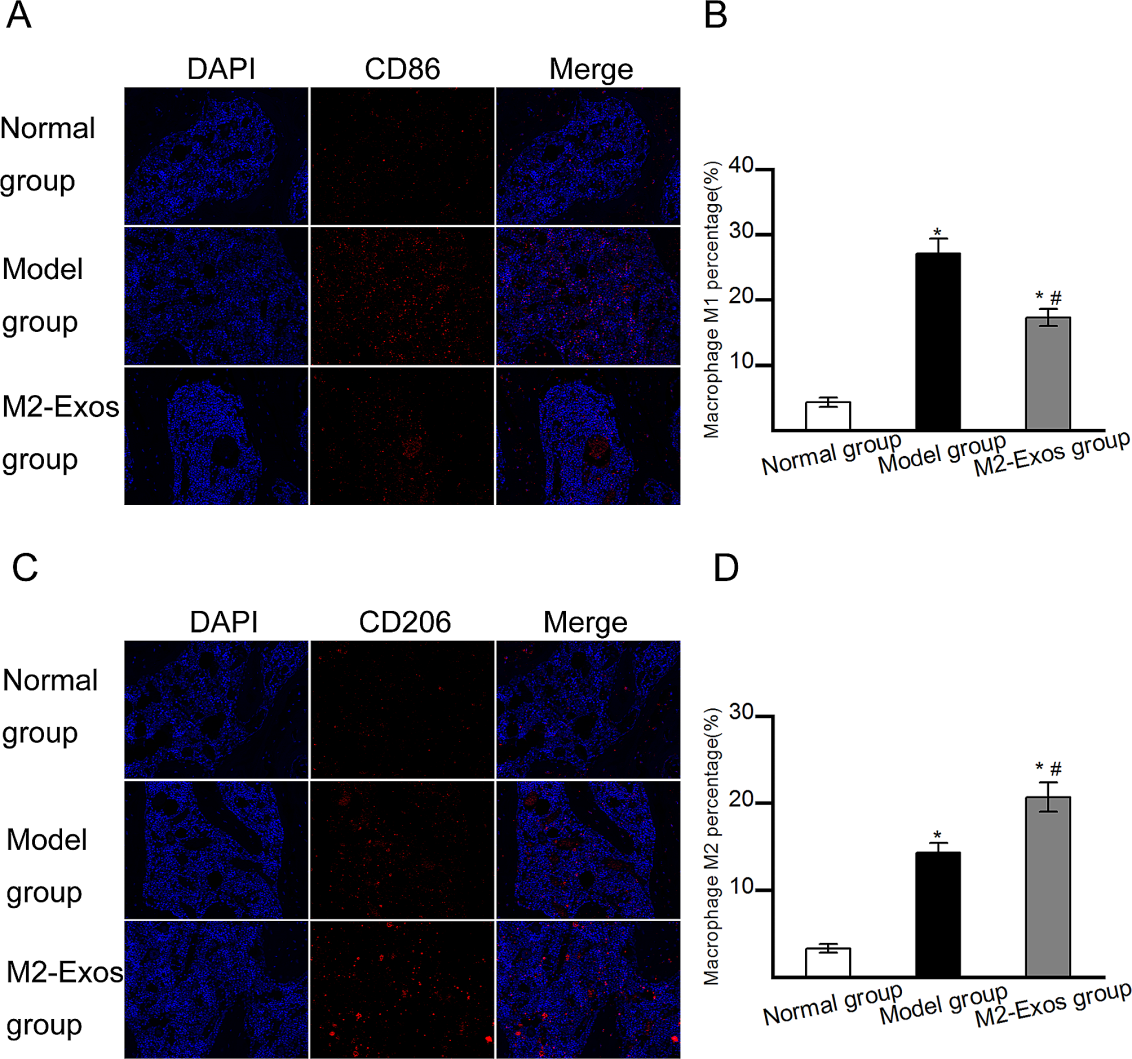
Infiltrations of M1/M2 macrophages in vivo. (A) M1 macrophages detected by immunofluorescence staining for CD86. (B) Quantitative analysis of CD86 + M1 macrophages. (C) M2 macrophages detected by immunofluorescence staining for CD206. (D) Quantitative analysis of CD206 + M2 macrophages. *N* = 5 per group. *p < 0.05 compared with Normal group; #*p* < 0.05 compared with Model group

The effects of M2-Exos on M1/M2 macrophage alterations were detected in vivo. Immunofluorescence staining was performed to identify M1 and M2 macrophages. CD86 + cells were considered as M1 macrophages, and CD206 + cells were considered M2 macrophages. As shown in Fig. 7, both the number of M1 macrophages and the number of M2 macrophages significantly increased in Model group compared with those in Normal group. However, the number of M1 macrophages significantly decreased and the number of M2 macrophages increased in M2-Exos group compared with those in Model group. The results indicated that the infiltration of both M1 and M2macrophages were enhanced in Model group compared with Normal group. M2-Exos treatmentinhibited the infiltration of M1 macrophages and promoted M2 macrophage polarization in the femoral head. The decreased M1 macrophages could be beneficial for resolving inflammation, and the increased M2 macrophages could be beneficial for accelerating tissue repair.

## Discussion

Exosomes are extracellular vesicles with a bilayer of phospholipids, which can transfer biological macromolecules such as proteins, nucleic acids and cytokines to recipients to influence the biological processes. Recently, some studies have shown that the exosomes derived from M2 macrophages(M2-Exos) display anti-inflammatory properties, can accelerate wound healing and play an important anti-inflammatory role in the inflammatory response in the nervous, digestive, respiratory and circulatory systems [20]. Yang R et al. reported that the exosomes of M2b macrophages upregulate the amount of Tregs, reduce the release of proinflammatory factors and exert an anti-inflammatory role in DSS-induced colitis in mice [21]. In a rat model of myocardial ischemia/reperfusion injury, it was found that exosomes derived from M2 macrophages deliver microRNA-148a and alleviate myocardial ischemia/reperfusion injury by inhibiting thioredoxin interacting protein (TXNIP) and the TLR4/NF-jB/NLRP3 inflammasome signalling pathway [22]. However, to the best of our knowledge, the role of M2-Exos in steroid-induced ONFH has not yet been reported. Considering the roles of M2-Exos in anti-inflammatory and immunoregulatory activity, we studied the effects of M2-Exos on steroid-induced osteonecrosis and explored the effect of M2-Exos on bone marrow-derived mesenchymal stem cells(BMMSCs) in vitro and the effect of M2-Exos on inflammation, the appearance of M1/M2 macrophages, bone resorption and bone formation (osteogenesis and angiogenesis) in vivo to identify the underlying mechanisms involved. The results showed that treatment with M2-Exos promoted the proliferation and osteogenic differentiation of BMMSCs. In addition, we found that M2-Exos effectively inhibited the expression of proinflammatory mediators, promoted osteogenesis and angiogenesis, reduced osteoclastogenesis and adipogenesis, and attenuated the osteonecrotic changes in steroid-induced ONFH.

Recently, several studies have investigated the crosstalk between M2 macrophages and bone marrow derived mesenchymal stem cells, and confirmed the regulation effect of exosomes derived from M2 macrophage on mesenchymal stem cells property. Several studies have reported that M2 macrophages can promote osteogenic differentiation of MSCs [33–35]. In addition, it was reported that conditioned media (CMs) generated by M2 macrophages facilitates BMMSCs osteogenic differentiation [36]. However, another study demonstrated that the exosomes secreted by M1 macrophages promote the proliferation and osteogenic differentiation of BMMSCs, and the exosomes secreted by M2 macrophages impair the proliferation of BMMSCs and show no influence on the osteogenic differentiation of BMMSCs [37]. It is inconsistent with our present study which proved that the exosomes derived from M2 macrophages promoted the proliferation and osteogenic differentiation of BMMSCs. More recently, a study by Zhou et al. indicated that exosomes from M2 macrophages promote the osteogenic differentiation of BMMSCs, which is consistent with our results [38]. This discrepancy can be attributed to the differences in cell lineage of macrophages and culture conditions.

Inflammation is involved in the pathogenesis of steroid-induced ONFH. Studies have shown that the levels of the proinflammatory cytokines TNF-α and IL-6 are predictably increased in the femoral head and in serum of individuals with steroid-induced ONFH [4, 5]. In our study, we got similar results. We observed that the levels of TNF-α and IL-6 dramatically increased in the local femoral head and in the circulation of rats with steroid-induced ONFH. Considering the important role of inflammatory reactions in steroid-induced ONFH, some studies have explored the therapeutic efficacy of inhibiting inflammatory reactions on ONFH. Wu X et al. studied the therapeutic potential of IL-4 in the prevention of steroid-induced osteonecrosis and reported that suppression of underlying inflammation by IL-4 alleviates steroid-associated osteonecrosis in mice by inhibiting the infiltration of M1 phenotypic macrophages and suppressing osteocytic apoptosis [10]. In a study of Kuroyanagi G et al., it was indicated that depletion of IL-6, a proinflammatory cytokine, is beneficial for bone healing following ischemic osteonecrosis in mice by stimulating revascularization and new bone formation [6]. In another study by Ren Y et al., IL-6 was inhibited by tocilizumab, and similar results were obtained [7]. They found that inhibiting IL-6 by tocilizumab can inhibit bone resorption and promote bone regeneration and vascular regeneration, thus promoting the repair of osteonecrosis. In our study, M2-Exos were administered to inhibit the inflammatory response. The expression of proinflammatory cytokines, including TNF-α and IL-6, was downregulated, and the osteonecrotic changes were attenuated with promoted osteogenesis and angiogenesis in rats with steroid-induced ONFH following treatment with M2-Exos.

Bone homeostasis is tightly controlled by osteoclast-mediated bone resorption and osteoblast-mediated bone formation. Inflammatory cytokines are reported to regulate the balance between osteoclasts and osteoblasts and are thought to be associated with the pathogenesis of ONFH [39]. Proinflammatory cytokines such as IL-1 and TNF-α cause an imbalance in bone metabolism by favoring bone resorption via the induction of RANKL and ICAM-1 [40]. Deletion of the proinflammatory cytokine IL-6 can stimulate osteogenesis during bone repair following osteonecrosis [6]. In our study, the model group showed increased proinflammatory mediators with promoted osteoclastogenesis and reduced osteogenesis, as indicated by quantitative analysis of micro-CT scanning, the mineral apposition rate and TRAP staining. After treatment with M2-Exos, the M2-Exos group showed decreased proinflammatory mediators with promoted osteogenesis and reduced osteoclastogenesis.

Macrophages are the sentinels of innate immunity. They respond to pathogens and other noxious stimuli, such as apoptotic or necroptotic cell debris, and initiate inflammatory responses [41]. Studies have indicated that macrophages also participate in the occurrence of steroid-induced ONFH [8]. Macrophages display different phenotypes depending on their environment, including classically activated M1 macrophages and alternatively activated M2 macrophages. M1 macrophages exhibit a proinflammatory phenotype and M2 macrophages exhibit an anti-inflammatory phenotype. Proinflammatory mediators such as IL-6 and TNF-α stimulate M1 macrophage polarization, while anti-inflammatory mediators such as IL-4 stimulate M2 macrophage polarization [42]. M1 macrophages contribute to increased bone resorption via the secretion of proinflammatory cytokines, and M2 macrophages play important roles in inducing osteogenesis [43, 44]. Tan Z et al. reported that the M1/M2 macrophage ratio in femoral heads from patients gradually increases from progressive-stage to end-stage nontraumatic osteonecrosis of the femoral head [45]. These findings indicate that M1/M2 macrophage imbalance facilitates the progression of nontraumatic ONFH and that switching macrophages to the M2 phenotype or inhibiting the M1 phenotype may be useful therapeutic strategies against nontraumatic ONFH. Similar to previously published studies [10, 46], the infiltration of both M1 macrophages and M2 macrophages was induced in the femoral heads of the model group. However, after treatment with M2-Exos, the femoral heads showed reduced M1 infiltration and further increased M2 infiltration, thus suppressing the inflammatory response and osteoclastogenesis, and facilitating osteogenesis.

The current study has potential limitations. The exact molecular mechanism underlying the preventive effects of exosomes derived from M2 macrophages in steroid-induced ONFH has not been investigated. Whether biological macromolecules in exosomes derived from M2 macrophages, such as microRNA, long noncodingRNA and functional proteins, participate in the therapeutic effects needs to be explored. In addition, the in vitro effects of exosome derived from M2 macrophages on inflammatory response and macrophage polarization are not investigated. Further clinical and basic studies are needed to address these limitations.

## Conclusion

In conclusion, this study showed that M2-Exos had anti-inflammatory effects and attenuated the osteonecrotic changes in steroid-induced ONFH by inhibiting the expression of proinflammatory mediators, regulating the polarization of M1/M2 macrophages, promoting osteogenesis and angiogenesis, and reducing osteoclastogenesis and adipogenesis. This study may be helpful for achieving greater resolution for steroid-induced ONFH.

## Data Availability

All relevant data are within the paper.

## Abbreviations

ALP: Alkaline phosphatase
ARS: Alizarin Red staining
BMD: bone mineral density
BMMSCs: bone marrow-derived mesenchymal stem cells
BV/TV: bone volume/total volume of bone
CCK-8: Cell Counting Kit-8 assays
DAPI: 4,6-diamino-2-phenylindole
EVs: extracellular vesicles
FBS: fetal bovine serum
LPS: lipopolysaccharide
M2-Exos: exosomes derived from M2 macrophages
MAR: mineral apposition rate
M-CSF: monocyte-colony stimulating factor
MPS: methylprednisolone
ONFH: osteonecrosis of the femoral head
p-NPP: p-nitrophenyl phosphate
PPAR-γ: osteocalcin(OCN) and peroxisome proliferators-activated receptorsγ
ROI: region of interest
RT-qPCR: Quantitative reverse transcription polymerase chain reaction analysis
Tb.N: trabecular number
Tb.Sp: trabecular separation
TRAP: tartrate-resistant acid phosphatase
VEGF: vascular endothelial growth factor
αMEM: α-minimum essential medium
